# Mathematical anxiety is linked to reduced cognitive reflection: a potential road from discomfort in the mathematics classroom to susceptibility to biases

**DOI:** 10.1186/1744-9081-10-31

**Published:** 2014-09-01

**Authors:** Kinga Morsanyi, Chiara Busdraghi, Caterina Primi

**Affiliations:** 1 School of Psychology, Queen’s University Belfast, David Keir Building, Belfast BT7 1NN, Northern Ireland, UK; 2 Department of NEUROFARBA, Section of Psychology, University of Florence, Florence, Italy

**Keywords:** Cognitive reflection test, Decision making, Dual-task manipulation, Heuristics and biases, Mathematical anxiety, Numeracy, Rationality, Test anxiety, Working memory

## Abstract

**Background:**

When asked to solve mathematical problems, some people experience anxiety and threat, which can lead to impaired mathematical performance (Curr Dir Psychol Sci 11:181–185, 2002). The present studies investigated the link between mathematical anxiety and performance on the cognitive reflection test (CRT; J Econ Perspect 19:25–42, 2005). The CRT is a measure of a person’s ability to resist intuitive response tendencies, and it correlates strongly with important real-life outcomes, such as time preferences, risk-taking, and rational thinking.

**Methods:**

In Experiments 1 and 2 the relationships between maths anxiety, mathematical knowledge/mathematical achievement, test anxiety and cognitive reflection were analysed using mediation analyses. Experiment 3 included a manipulation of working memory load. The effects of anxiety and working memory load were analysed using ANOVAs.

**Results:**

Our experiments with university students (Experiments 1 and 3) and secondary school students (Experiment 2) demonstrated that mathematical anxiety was a significant predictor of cognitive reflection, even after controlling for the effects of general mathematical knowledge (in Experiment 1), school mathematical achievement (in Experiment 2) and test anxiety (in Experiments 1–3). Furthermore, Experiment 3 showed that mathematical anxiety and burdening working memory resources with a secondary task had similar effects on cognitive reflection.

**Conclusions:**

Given earlier findings that showed a close link between cognitive reflection, unbiased decisions and rationality, our results suggest that mathematical anxiety might be negatively related to individuals’ ability to make advantageous choices and good decisions.

## Background

Although children begin their formal education with a very positive view of mathematics [1,2], as they progress through education, many of them develop negative feelings and attitudes (cf., [3]). The feeling of tension, apprehension and fear experienced when faced with maths content has been termed mathematical anxiety [4]. Maths anxiety interferes with mathematics performance and it leads to negative maths-related attitudes and self-perceptions, an avoidance of elective maths courses, and it also affects people’s career opportunities and career choices [5]. Whereas the negative consequences of maths anxiety on educational outcomes are well-known, it is less clear whether maths anxiety also affects people’s behaviour in other contexts.

It is known that, at the population level, low numeracy is associated with reduced economic productivity [6], higher rates of unemployment, mental and physical illness, as well as higher rates of arrest and incarceration [7]. Low numeracy also impairs risk comprehension, and leads to biases in judgment and decision making (see [8,9] for reviews). Nevertheless, the link between low numeracy and maths anxiety is not so clear-cut. Several studies investigated the effects of maths anxiety in educational contexts. Although correlations of around -.30 have been reported between mathematics anxiety and mathematics achievement (see [5,10] for meta-analyses), these correlations might be inflated, because mathematics achievement in these studies is typically based on measures collected in high-stakes test situations, which are especially likely to induce anxiety (cf., [11]). Other studies (e.g., [12]) found a link between maths anxiety and arithmetic performance in experimental settings. Although these findings suggest that maths anxiety can lead to suboptimal maths performance, it is not the case that maths anxiety is necessarily associated with low levels of numeracy or poor maths performance (cf., [13,14]).

Lab-based investigations have revealed the cognitive mechanisms underpinning the negative effects of maths anxiety. When solving arithmetical problems, participants with high maths anxiety have higher error rates, they have problems with rejecting incorrect solutions (even when these are highly implausible), and these effects are especially prominent in the case of more complex problems which require a carry operation (e.g., [15,16]). Participants with high maths anxiety also often show a characteristic pattern of responding quickly to problems, displaying a “local avoidance” of the uncomfortable test situation (cf., [13,16]), and probably decreasing their chances of generating correct responses. Studies have also indicated that maths anxiety has an online effect on maths performance through hampering working memory resources [12,17].

It is easy to imagine how maths anxiety might emerge in, and impact on some everyday situations, such as estimating the price of purchases, splitting the bill in a restaurant, choosing between electricity suppliers, or deciding on investment options. For example, Silk and Parrott [18] reported that participants’ maths anxiety increased after they were presented with statistical information about the health risks associated with consuming genetically modified food. These authors also reported a negative association between maths anxiety and numeracy, as well as between maths anxiety and the ability to interpret health statistics. Nevertheless, the effect of maths anxiety on the everyday functioning of individuals, and, in particular, their ability to make good decisions is unknown. Thus, the aim of the following studies was to explore the link between mathematical anxiety and a short test which was found to be highly predictive of individuals’ decision-making skills: the Cognitive Reflection Test (CRT; [19]). Additionally, we were also interested in whether the link between maths anxiety and cognitive reflection is also present when participants’ maths knowledge and maths achievement are taken into account. In order to investigate these questions, in Experiment 1, we used a test of basic mathematical knowledge, which was specifically developed for university students [20]. This test can also be considered as a measure of numeracy, and it was administered in a low-stakes situation, as part of the research study. In Experiment 2, participants’ final secondary school maths grades were used. This was intended as an ecologically valid indicator of maths achievement in an academic setting.

The CRT [19] is a short test measuring a person’s tendency to override an intuitively compelling response, and to engage in further reflection which can lead to a correct solution. Although the CRT was not intended as a decision-making test *per se*, but as a “simple measure of one type of cognitive ability” ([19], p. 26), both Frederick [19] and others (e.g., [21-23] demonstrated that it was highly predictive of performance on tests of decision-making ability. As an example, consider the following item:

A bat and a ball cost $1.10 in total. The bat costs $1.00 more than the ball. How much does the ball cost? ______ cents

Although the correct response is 5 cents, many participants give the response “10 cents”, which seems to pop into mind effortlessly. Cognitive reflection involves the ability to effectively monitor and correct impulsive response tendencies.

Cognitive reflection was found to be negatively related to temporal discounting (i.e., the tendency to prefer smaller, immediately available rewards over larger rewards which will be available later), and positively related to choosing gambles with higher expected values [19]. Further studies showed that the CRT was also related to some typical heuristics and biases [21-23]. One could argue that these relationships are unsurprising, given that the CRT is based on mathematical word problems, and many of these judgment and decision-making tasks also contain numerical information. However, the CRT was also related to tasks containing no numerical information (for example, syllogistic reasoning, which measured the effect of beliefs on logical reasoning; see [23]). Furthermore, although the CRT correlates with measures of intelligence and numeracy (e.g., [19,24]) it was found to explain additional variance in reasoning and decision-making tasks when it was administered together with measures of intelligence and numeracy [21,23,25]. Overall, these results demonstrate that the CRT is a very powerful predictor of a person’s ability to make unbiased judgments and rational decisions on a variety of tasks.

In sum, in the studies that we report below we tested the hypothesis that maths anxiety might be related to performance on the CRT. We based this prediction on the observation that maths anxiety leads to a tendency to generate and accept responses quickly, without taking the opportunity to check solutions and correct mistakes, and to accept incorrect responses even when these are highly implausible (cf., [16]). In other words, it seems that maths anxiety reduces cognitive reflection.

It could be argued that a potential link between maths anxiety and cognitive reflection would be unsurprising, given that the CRT problems contain numerical content. Thus, we also aimed at showing that even when the effect of mathematical abilities or mathematical achievement is controlled for, the link between maths anxiety and cognitive reflection would still be present. We based this prediction on earlier claims that the CRT is “not just another numeracy scale” (cf., [21]; p.361), but also a potent indicator of a person’s ability to avoid tempting, easy-to-generate heuristic responses [19]. This prediction is also in line with Campitelli and Gerrans [25] who found that the ability to inhibit the effect of beliefs on logical reasoning explained additional variance in the CRT, when the effects of numeracy were already taken into account. The prediction that the link between the CRT and maths anxiety is not fully mediated by maths knowledge or maths achievement is key to our account, as we aim to show that mathematical anxiety is negatively related to individuals’ ability to avoid the typical pitfalls of reasoning and decision-making.

Additionally, in line with a recent study [26] we wanted to control for the effects of test anxiety. This is important, as maths anxiety and test anxiety show moderate correlations, and it has been argued that maths anxiety could be just a form of test anxiety ([27], although see [28]).

In summary, in the studies that we report below, we tested the prediction that maths anxiety is linked to relatively poor performance on the CRT, even when the effects of general mathematical knowledge or school maths achievement, and test anxiety are taken into account. We tested this prediction in two populations: secondary school and university students. In our final study we also investigate the cognitive mechanisms behind the link between maths anxiety and cognitive reflection.

## Experiment 1

In Experiment 1 we tested the prediction that maths anxiety is negatively related to cognitive reflection. Specifically, we tested a path model in which mathematical anxiety is directly related to poor performance on the CRT. Additionally, we hypothesized an indirect link between maths anxiety and cognitive reflection through general mathematics knowledge. Finally, we also tested the potential effect of test anxiety on these links.

### Method

#### Participants

The participants were 328 female psychology students (*M*_age_ = 20.8; *SD* = 3; range 18 to 53 years) from the University of Florence, Italy. ^a^ All students participated on a voluntary basis, and they were offered ungraded course credit for their participation.

#### Materials

*Cognitive reflection test* (CRT; [19]): The CRT is a three-item test which consists of word problems including numerical information (see an example item above). A single composite score was calculated, based on the sum of correct responses. We administered an Italian version of the items. In this version, the task that we described above was modified in the following way. We included euros instead of dollars, and the bat and the ball were changed to a chocolate bar and a piece of candy. The other two items were translated into Italian without any modification of the content. In the present sample, Cronbach’s alpha was .57.

*Mathematics Prerequisites for Psychometrics* (MPP; [20]): This test was developed with the aim of measuring the mathematics skills needed by students enrolling in introductory statistics courses. The scale was constructed applying item response theory analyses, and its reliability and validity were tested. The test consists of 30 problems, and it has a multiple-choice format (one correct response out of four alternatives). A single composite, based on the sum of correct responses, was calculated. In the present sample.

Cronbach’s alpha was .80. We used this measure as an estimate of students’ maths knowledge.

*Abbreviated Maths Anxiety Scale* (*AMAS*, [29]; see details on the Italian adaptation of the scale in [30]). This 9-item scale measures maths anxiety experienced by students in learning and test situations. High scores indicate high levels of anxiety. Participants have to respond on the basis of how anxious they would feel during the events specified, using a 5-point response scale (ranging from *1 = low anxiety* to *5 = high anxiety*). An example item is: *“Thinking about an upcoming math test one day before”.* A single composite score was obtained, based on participants’ ratings of each statement. Previous studies showed that the AMAS has satisfactory reliability and validity [29,31]. In the present sample Cronbach’s alpha was .86.

Test Anxiety Inventory (TAI, [32]). The scale contains 20 items designed to measure anxiety associated with test-taking situations. Reliability coefficients, and convergent validity with other measures of test anxiety are strong [33]. Participants have to respond on the basis of how they would generally feel during the events specified using a 4-point response scale (ranging from *1 = almost never* to *4 = almost always*). An example item is: *“Thinking about my grade in a course interferes with my work on tests”.* The Italian version of the TAI was obtained from the English version using the *forward-translation* method. Two non-professional translators worked independently, and then they compared their translations with the purpose of assessing equivalence. Subsequently, a group of five people read this first version, revised it, and eventually obtained a final form. A single composite score was obtained, based on participants’ ratings of each statement. In our sample Cronbach’s alpha was .93.

#### Procedure

The scales were presented in a paper-and-pencil format, and students participated in groups of around 40. They worked through the CRT and the questionnaires individually. The order of presentation was: MPP, TAI, AMAS and CRT. Participants were informed that participation was voluntary and anonymous, and they were asked to give written informed consent. The average administration time was 50 minutes.

### Results

Table 1 presents the descriptive statistics for our measures, and the correlations between them. As expected, maths anxiety and maths knowledge were both significantly correlated with cognitive reflection, and maths anxiety was also significantly (negatively) correlated with maths knowledge. Additionally, mathematical anxiety was also related to test anxiety.

**Table 1:** Correlations between cognitive reflection, maths knowledge, and maths and test anxiety, and descriptive statistics for each measure

	**1**	**2**	**3**	**4**
1. Cognitive reflection	–			
2. Maths knowledge	.45***	–		
3. Maths anxiety	-.20***	-.17**	–	
4. Test anxiety	-.05	-.05	**.**54***	–
*M* (*SD*)	.90 *(.94)*	22.6 *(4.5)*	24.1 *(6.5)*	43.6 *(11.8)*

**p < .01; ***p < .001.

**Figure 1:**
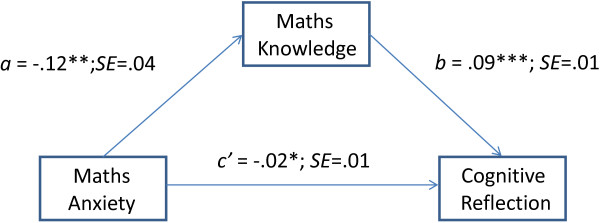
Path model depicting the relationships between maths anxiety, maths knowledge, and cognitive reflection (a, b, c’ are unstandardized OLS regression coefficients);* p < .05 **p < .01; ***p < .001).

To better understand the mechanism underlying the relationships between these variables, we tested the hypothesis that maths knowledge mediates the effect of maths anxiety on cognitive reflection (see Figure 1). Recently, researchers [34,35] have recommended the use of the bootstrapping procedure over the Sobel test in assessing indirect effects of mediation models, since the bootstrapping procedure does not impose the assumption of normality of the sampling distribution on indirect effects. In our study, the mediaton hypothesis was tested with SPSS macro (the INDIRECT procedure) for bootstrapping (with 5000 bootstrap samples) to estimate 95% confidence interval (CI; for more details, see Preacher and Hayes [36]).

We estimated the indirect effect of maths anxiety on cognitive reflection, quantified as the product of the Ordinary Least Squares (OLS) regression coefficient estimating maths knowledge from maths anxiety (path *a* in Figure 1), and the OLS regression coefficient estimating cognitive reflection from maths knowledge controlling for math anxiety (path *b* in Figure 1). A bias-corrected bootstrap-confidence interval (CI) of the product of these paths that does not include zero provides evidence of a significant indirect effect of maths anxiety on cognitive reflection through maths knowledge [36]. The INDIRECT procedure revealed a significant indirect effect of maths anxiety on cognitive reflection through maths knowledge (point estimate - .01, 95% *CI* = - .02 to - .004). Kappa squared was 0.074 (95% *CI* = .026, to .128) indicating a medium effect size for the indirect effect [37].

Figure 1 also shows the significant parameter associated with path *c’* which indicates a direct effect of maths anxiety on the CRT. More specifically, participants with high maths anxiety were less likely to give a correct answer to the CRT items. In sum, the results show that, when the effect of maths knowledge on cognitive reflection was controlled for, the link between maths anxiety and cognitive reflection was still significant. Overall, the results indicate that the link between maths anxiety and the CRT was partially mediated by mathematical knowledge, but even when this effect was controlled for, there was a direct link between maths anxiety and the CRT. Finally, we investigated whether these effects were affected by test anxiety. We included test anxiety as a covariate in the mediation model simultaneously with the other predictor variables. Controlling for the effects of this covariate did not substantially change the relationships between maths anxiety, maths knowledge and cognitive reflection, and the effect of test anxiety was not significant (*p* = .30).

### Discussion

In Experiment 1 we tested the prediction that maths anxiety is negatively linked to cognitive reflection, and that this effect is present even when the potential indirect effects of maths anxiety through maths knowledge are controlled for. We expected this on the basis of earlier findings which showed that maths anxiety leads to a tendency to impulsively accept incorrect responses, without much reflection, at least in the case of arithmetic problems (cf., [16]). We also tested the potential effects of test anxiety on the links between maths anxiety, maths knowledge and cognitive reflection.

The results were in line with our predictions. Specifically, we have found both a direct link between maths anxiety and cognitive reflection, and an indirect link through maths knowledge. These results have several implications. One is that the effects of maths anxiety are not only present in the case of arithmetical operations which involve high computational demands, and are presented in the form of numbers and symbols, but also in the case of word problems with numerical content, where mathematical symbols are not used. Indeed, to the best of our knowledge, our study was the first to test the effect of maths anxiety on the ability to solve maths word problems. Nevertheless, our aim was not simply to demonstrate this link. Indeed, the CRT is considered to be more than just a maths task (cf. [21]). We expected, and found, that the link between maths anxiety and cognitive reflection was not simply the consequence of lower numeracy levels, or a reduced ability to apply maths knowledge in highly maths anxious individuals. Indeed, when we controlled for the effect of maths knowledge (as measured by the MPP), the link between maths anxiety and cognitive reflection was still significant. Overall, our results suggest that maths anxiety is linked to performance on the CRT in two ways: directly, and through an association with reduced maths knowledge.

Nevertheless, a weakness of our method is that we assessed maths knowledge as part of the experimental session. It could be argued that given the low-stakes situation, the effect of maths anxiety on maths knowledge was reduced, which could have decreased our chances of detecting a full mediation effect of maths knowledge between maths anxiety and cognitive reflection. We addressed this issue in Experiment 2.

## Experiment 2

In order to obtain a more ecologically valid measure of maths performance in a high-stakes situation, in Experiment 2 we included a measure of school maths achievement instead of a measure of maths knowledge. The other purpose of our study was to extend our investigation to a different population: secondary school students.

### Method

#### Participants

184 secondary school students participated in the study (*M*_age_ = 15.9; *SD* = .80; 15–19 years; 47% females). The participants were recruited from four secondary schools in a suburban area in Italy (Tuscany). A detailed study protocol that explained the study’s goal and methodology was approved by the institutional review boards of each school. Students received an information sheet, which assured them that the data obtained from them would be handled confidentially and anonymously, and they were asked to give written informed consent. Parents of minors were required to provide consent for their child’s participation. All the youth invited to participate agreed to do so.

#### Materials and procedure

As in the previous study, the Italian versions of the CRT, the AMAS, and the TAI were administered. Once again, all measures displayed good reliability (Cronbach’s alphas for the AMAS, TAI and CRT were .86, .93 and .68, respectively). As an indicator of maths achievement, the final secondary school maths grades were used (range: 0–10). The scales were presented in a paper-and-pencil format, and were administered to students in a classroom setting during school time. Students were provided with a brief introduction to the study, and with some instructions on how to complete the questionnaires. The order of presentation was the same as in Study 1.

### Results

Table 2 presents the descriptive statistics for our measures, and the correlations between them. As in Experiment 1, maths anxiety and maths achievement were both significantly correlated with cognitive reflection, and maths anxiety was also significantly (negatively) correlated with maths achievement. The relationship between maths and test anxiety was also significant.

**Table 2:** Correlations between cognitive reflection, maths achievement, and maths and test anxiety, and descriptive statistics for each measure

	**1**	**2**	**3**	**4**
1. Cognitive reflection	–			
2. Maths achievement	.29***	–		
3. Maths anxiety	**-**.20**	**-**.22**	–	
4. Test anxiety	.03	-.06	.49***	–
*M* (*SD*)	.64 *(.96)*	6.4 *(1.3)*	23.1 *(7.9)*	47.01 *(13.7)*

**p < .01; ***p < .001.

We tested the mediation hypothesis using the INDIRECT [36] procedure with 5,000 bootstrap samples. The results revealed a significant indirect effect of maths anxiety on cognitive reflection through maths achievement (estimate point -.007, 95% *CI* = - 0.014 to - 0.002). Kappa squared was .056 (95% CI = .017, to .112) indicating a medium effect size for the indirect effect [37].

**Figure 2:**
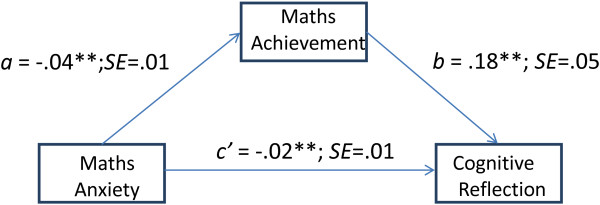
Path model depicting the relationships between maths anxiety, maths achievement, and cognitive reflection (a, b, c’ are unstandardized OLS regression coefficients; *p < .05;**p < .01).

The parameter associated with path *c’* was also significant, indicating a direct effect of maths anxiety on the CRT. Overall, these results show that, once the mediating effect of maths achievement was controlled for, the effect of maths anxiety on cognitive reflection was still significant.To further investigate whether these effects were affected by gender and test anxiety, we - simultaneously with all other predictor variables- included them in the mediation model. Controlling for the effect of these covariates did not substantially change the relationships between maths anxiety, maths knowledge and cognitive reflection. We also tested whether the covariates had a significant effect on cognitive reflection, and found that all of these effects were non-significant [Figure 2].

### Discussion

The aim of Experiment 2 was to extend our investigation to a different population, whilst we also used a more ecologically valid indicator of maths performance in high-stakes situations (i.e., course performance). The results confirmed our initial findings: maths anxiety predicted cognitive reflection, even when the effects of maths achievement and test anxiety were controlled for.

Whereas these results clearly demonstrate that there is a reliable association between maths anxiety and cognitive reflection, the causal link between these constructs is unclear. We hypothesized that maths anxiety led to a reduction in cognitive reflection. Nevertheless, in theory, it is also possible that reduced cognitive reflection leads to increased maths anxiety. Thus, one aim of our final study was to better understand the link between maths anxiety and cognitive reflection.

Ashcraft and Kirk [12] demonstrated that the effects of maths anxiety arose from the burden that anxious thoughts imposed on WM. Specifically, they showed that when participants’ WM was burdened by a secondary task, the effects of maths anxiety were especially apparent in the case of addition problems which included a carry operation (e.g., 17 + 25). That is, it appeared that the WM-load imposed by anxious thoughts, the carry operation and the secondary task added up, and disproportionately affected anxious participants’ performance, as compared to non-anxious participants, whose performance declined to a smaller degree under a dual-task load.

Other lines of research [38,39] demonstrated that in the case of tasks which involved a conflict between a heuristic and a normatively correct response, employing a dual-task load decreased normative performance, and increased participants’ tendency to give a heuristic response. Given that the CRT problems involve a heuristic-normative conflict, we expected that a dual-task load will lead to a reduction in normative responding. This prediction is also in line with Toplak et al. [23] who found a correlation between working memory capacity and performance on the CRT.

In summary, in Experiment 3 we tested the hypothesis that the relationship between maths anxiety and performance on the CRT was similar to the relationship between available WM capacity and performance on the CRT.

## Experiment 3

**Figure 3:**
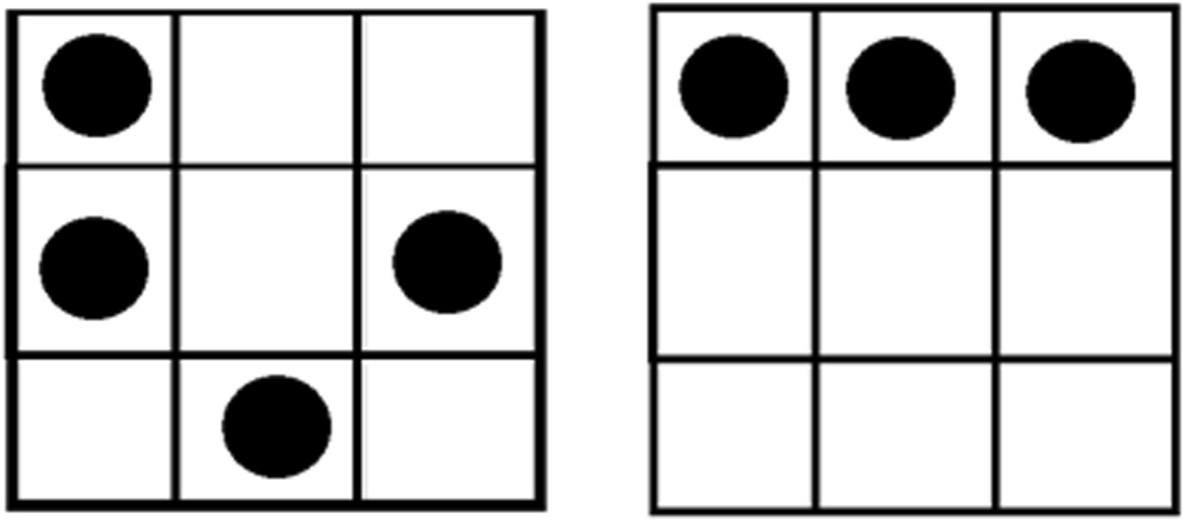
Examples of the dot patterns used in the high and low load conditions.

In our last experiment, we adopted a procedure which was previously used by De Neys [38], and Gillard et al. [39]. In these studies, participants had to memorize either simple or complex dot patterns (see Figure 3). That is, participants were allocated into either a low or a high WM-load condition. Additionally, De Neys [38] also included a control condition with no WM-load. In both studies, besides remembering the dot patterns, the participants were also asked to solve problems with a conflict between a tempting heuristic response and a normative response. We adopted the design used by De Neys [38] in the present study.

As we described above, in the case of tasks with a conflict between a heuristic and a normatively correct response, it has been demonstrated that burdening participants’ WM with a secondary task leads to an increase in heuristic and a reduction in correct responding [38,39]. The CRT is a task with conflicting heuristic and correct responses, and a previous study demonstrated that performance on the CRT was linked to WM-capacity [23]. Based on the idea that anxiety burdens WM resources (e.g., [12]), we hypothesized that WM load and anxiety will be linked to reasoning performance in a similar way. That is, we expected that both WM-load and maths anxiety would be linked to poor performance on the CRT.

Besides their relationship with reasoning accuracy, we were also interested in how WM-load and maths anxiety were related to reaction times on the CRT, participants’ evaluations of their performance (that is, their estimate of how many of the CRT problems they were able to solve correctly, after completing the tasks), and their self-reported anxiety that they experienced while completing the tasks. The purpose of including these additional measures was to better understand the mechanisms through which anxiety and working memory load are linked to cognitive reflection. Whereas there are good reasons to expect that anxious participants will rush through the tasks (cf., [13,16]), show lower confidence in their performance (e.g., [40]), and report higher levels of anxiety while solving the CRT problems, WM-load in itself might not be associated with such changes. In addition, including a measure of anxiety that participants experienced while they worked through the problems made it possible to assess the ecological validity of the AMAS with regard to solving the CRT problems.

Finally, in Experiments 1 and 2, in our path models we assumed that the negative relationship between mathematical anxiety and cognitive reflection arose because mathematical anxiety reduced cognitive reflection. Nevertheless, in theory, it is also possible that reduced cognitive reflection leads to increased maths anxiety. If this was the case, the WM-load manipulation (which is expected to reduce cognitive reflection) should lead to an increase in participants’ levels of anxiety while they are working through the tasks.

### Method

#### Participants

Eighty-nine students from a UK university participated in the study (66 females; *M*_age_ = 22 years 4 months; *SD* = 8.11; range: 18–56 years). The participants were recruited from undergraduate and postgraduate psychology courses, and they were either offered ungraded course credit or a small payment for participation. 31 participants (8 males) were allocated to the control group, 29 participants (8 males) were allocated to the low load, and 29 participants (7 males) were allocated to the high load condition.

#### Materials and procedure

##### Measures of mathematical and test anxiety

Maths anxiety was assessed by the AMAS (Cronbach’s alpha: .92), and test anxiety was assessed by the TAI (Cronbach’s alpha: .96).

##### Long version of the Cognitive Reflection Test

In this experiment we administered an 8-item version of the CRT. We developed this version in order to be able to discriminate between individual’s cognitive reflection skills with higher precision, and to reduce the proportion of participants who score 0 on the test. The items in this long version included the 3 original CRT items. 5 more items were added, which included 3 items based on modified versions of additional CRT items developed by Frederick (these items were not published previously), one item based on a modification of a task used by Gillard et al. [39], and an additional item developed by us. These items were obtained through an iterative process, whereby items with sub-optimal properties were modified or removed from initial longer versions of the scale. The eventually obtained 8-item version of the CRT was pre-tested on a sample of 560 undergraduate students. This pre-testing confirmed that (similarly to the original CRT test) the vast majority of participants generated either the typical heuristic (61% of responses) or the correct response (33% of responses) to these items. The scale was highly reliable (Cronbach’s alpha: .78). Detailed analyses of the psychometric properties of the scale are reported in Primi, C., Morsanyi, K., Donati, M., Chiesi, F.: *The development and testing of a new version of the Cognitive Reflection Test applying Item Response Theory (IRT).* in submission.

In the present study the 8-item version of the CRT was presented on the computer with the modification that participants were not asked to generate responses, but they had to select from three response options: the typical heuristic response, the correct response, and an atypical response. The atypical response was chosen to be dissimilar from both other responses, and it was also a response that no participants had spontaneously generated during the pre-testing of the items. We chose a forced choice format, because we wanted to minimize the number of incorrect responses stemming from arithmetical errors (as opposed to incorrect mathematical reasoning). Given that correct response rates on the CRT are generally low (around 30%), and that we administered the problems together with a secondary task, it was important to avoid a floor effect. Cronbach’s alpha in the present experiment was .69.

The *secondary task* was the Dot Memory Task (DMT), a classic spatial storage task previously used by De Neys [38] and Gillard et al. [39]. A 3x3 matrix, filled with three or four dots was presented (see Figure 3). Participants had as much time as they needed to memorize the pattern. Then they solved a CRT problem, and they had to recall the dot pattern afterwards by clicking on the appropriate cells of the matrix. In the high load condition, the matrix was filled with a complex four-dot pattern (i.e., a “three-piece” pattern based on Bethell-Fox and Shepard [41]). Miyake et al. [42] established that storage of similar complex dot patterns burdens executive resources. In the low load condition, the pattern consisted of three dots on a horizontal line (i.e., a “one-piece” pattern) which burdens executive resources to a smaller degree. In order to draw attention to the importance of the secondary task, participants were provided with feedback (i.e., correct/incorrect) each time they recalled the position of the dots. Participants in the control condition worked through the CRT problems without performing a secondary task.

The AMAS and TAI were presented in a paper-and-pencil format. The CRT was presented on the computer, together with the DMT. Both tasks were explained to participants, and they solved a practice problem before working through the test problems. For the CRT, both accuracy and reaction times were recorded. Immediately after completing the CRT, participants were asked to report the level of anxiety that they experienced while working through the CRT problems on a 10-point scale (ranging from *1 = no anxiety at all*; to *10 = very high levels of anxiety*). Participants were also asked to give an estimate of how many out of the 8 tasks they solved correctly. The average testing time was 35 minutes.

### Results

Accuracy on the DMT was high in both the low load (91%) and in the high load (93%) conditions. That is, participants engaged in the secondary task fully. Preliminary analyses suggested that low and high WM-load affected accuracy and reaction times on the CRT to a similar degree, and, thus, we report the results collapsed across the two WM-load conditions. We also checked the proportion of atypical (i.e., neither heuristic nor correct) responses on the CRT tasks. Atypical response rates were extremely low (3.5%). That is, as expected, participants primarily selected the heuristic and correct responses.

As a preliminary step for our main analyses, we created high and low maths anxiety groups by performing a median split, with participants with an AMAS score of 20 or below allocated to the low anxiety group (*n* = 46).

**Figure 4:**
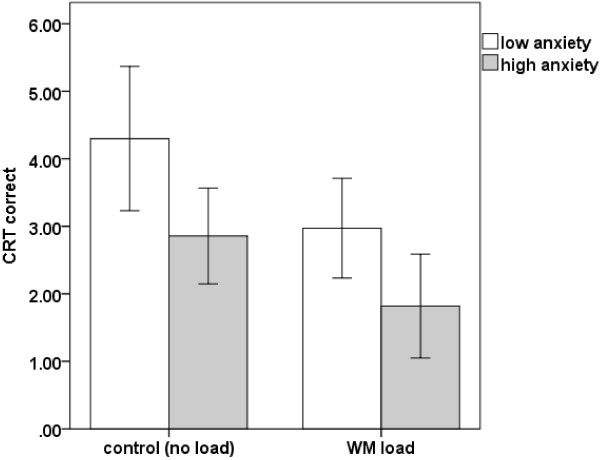
The effects of maths anxiety and cognitive load on performance on the cognitive reflection tasks (error bars represent 95% confidence intervals).

The purpose of our first analysis was to test the effects of cognitive load and mathematical anxiety on cognitive reflection. A 2x2 ANOVA with maths anxiety group (high/low) and WM load (WM load/no load) as between-subjects factors indicated a significant effect of maths anxiety (*F*(1,88) = 8.68, *p* = .004, *η*_*p*_^*2*^ = .09), and a significant effect of WM load (*F*(1,88) = 7.21, *p* = .009, *η*_*p*_^*2*^ = .08). There was no interaction between anxiety and cognitive load (*p* = .74). Figure 4 demonstrates these effects.

In order to demonstrate that the effect of anxiety was specific to mathematical anxiety, and was not a side-effect of general test anxiety, we replicated the previous analysis including test anxiety as a covariate. The effect of test anxiety was not significant (*p* = .60). However, the effects of maths anxiety (*F*(1,88) = 5.07, *p* = .027, *η*_*p*_^*2*^ = .06), and WM load (*F*(1,88) = 7.36, *p* = .008, *η*_*p*_^*2*^ = .08) remained significant. As in the previous analysis, there was no interaction between anxiety and cognitive load (*p* = .66).

Our next analysis investigated the effects of maths anxiety and WM load on reaction times on the CRT. A 2x2 ANOVA with maths anxiety group (high/low) and WM load (WM load/no load) conditions as between-subjects factors indicated a significant effect of maths anxiety (*F*(1,88) = 5.01, *p* = .028, *η*_*p*_^*2*^ = .06), but no significant effect of WM load (*p* = .93), and no interaction between WM load and anxiety (*p* = .42). Highly anxious participants spent on average 17.31 seconds (*SD* = 5.08) on solving each problem, whereas participants with low maths anxiety spent 22.86 seconds (*SD* = 17.31) on average on each problem. However, WM-load did not have an effect on the time that participants took to solve each problem ^b^.

We also checked the effects of maths anxiety and WM load on self-reported anxiety levels while working through the problems. This was important in establishing that our measure of general maths anxiety was actually related to the anxiety of participants while they worked through the cognitive reflection items. Note that measures of maths anxiety are typically used in classroom settings, or in relation to arithmetic problem solving. In our study the CRT tasks were not explicitly presented as maths problems, and participants performed the tasks anonymously, which reduced performance pressure.

A 2x2 ANOVA with maths anxiety group (high/low) and WM load (WM load/no load) conditions as between-subjects factors indicated a significant effect of maths anxiety (*F*(1,88) = 32.88, *p* < .001, *η*_*p*_^*2*^ = .28), but no significant effect of WM load (*p* = .58), and no interaction between the two factors (*p* = .91). Highly anxious participants’ average self-reported anxiety level was 4.91 (*SD* = 2.19) on a 10-point scale (indicating moderate levels of anxiety), while the average rating of participants with low AMAS scores was 2.52 (*SD* = 1.36), which reflected low levels of experienced anxiety.

We also computed the correlations between self-reported anxiety levels and participants’ AMAS and TAI scores. The correlation between mathematical anxiety and self-reported anxiety was *r*(86) = .56, *p* < .001, whereas the correlation between self-reported anxiety and test anxiety was *r*(86) = .36, *p* < .001. Importantly, the correlation between mathematical anxiety and self-reported anxiety was still significant after controlling for the effect of test anxiety *r*(85) = .45, *p* < .001. By contrast, after controlling for the effect of mathematical anxiety, there was no relationship between test anxiety and self-reported anxiety while solving the CRT problems (*p* = .78).

In our final analysis, we checked the effects of maths anxiety and WM-load on students’ estimates of how many CRT items they solved correctly. A 2x2 ANOVA with maths anxiety group (high/low) and WM load (WM load/no load) conditions as between-subjects factors indicated a significant effect of maths anxiety (*F*(1,88) = 10.02, *p* = .002, *η*_*p*_^*2*^ = .11), but no significant effect of WM load (*p* = .11), and no interaction between WM-load and anxiety (*p* = .55). Highly anxious participants expected that they solved 3.43 items correctly (*SD* = 1.32), and participants with low levels of anxiety expected that they solved 4.34 items correctly (*SD* = 1.68). In fact, participants in both groups overestimated their performance (correct response rates were *M* = 3.26, *SD* = 2.11 for low anxiety, and *M* = 2.33, *SD* = 1.71 for high anxiety participants).

### Discussion

In Experiment 3 the link between maths anxiety and performance on the CRT was investigated together with the effect of WM-load on performance on the CRT. In line with our hypotheses, we found that both anxiety and WM load were associated with relatively poor performance on the CRT. The effects of WM load were similar in the case of participants with high and low anxiety (i.e., anxiety and WM load did not interact). These results are in line with earlier claims that anxious thoughts regarding mathematical content act in a similar way as WM-load [12,17] insofar as both WM-load and anxiety are associated with performance decrements.

At the same time, our results also demonstrate that the link between mathematical anxiety and cognitive reflection did not simply arise due to the working memory load imposed by anxious thoughts. Indeed, working memory load in itself did not lead to reduced response times (i.e., rushing through the problems), or to reduced levels of confidence in one’s own performance. The latter finding is especially interesting, given that performance actually deteriorated under cognitive load.

Overall, these results suggest that mathematical anxiety is associated with lower performance, reduced confidence, and faster responses. It is also clear that the reduced availability of working memory resources does not result in reduced confidence or shorter reaction times. Nevertheless, further investigations are needed to establish whether there is a causal relationship between reaction times and confidence levels. For example, one possibility would be that participants rush through the problems, because they do not trust in their ability to solve them, and, thus, they do not expect that investing more time would improve their performance. In the present study there was a weak, but significant correlation between self-estimated performance and reaction times (*r*(87) = .23, *p* = .029). ^c^However, once we controlled for the effect of self-reported anxiety during the testing session, this relationship disappeared (*p* = .18). Thus, the effects associated with maths anxiety (i.e., reduced confidence and shorter reaction times) might arise independently of each other.

Another interesting finding is that although WM-load reduced cognitive reflection, it did not lead to increased levels of self-reported anxiety. This suggests that whereas increased anxiety is linked to reduced cognitive reflection, a manipulation of cognitive reflection does not affect anxiety levels. This is in line with the interpretation that maths anxiety reduces cognitive reflection. Nevertheless, given that anxiety was not manipulated in our experiment, we are unable to make strong causal claims. Indeed, it is possible that a common underlying factor affects anxiety levels and performance on the CRT.

As we described above, we found a negative association between maths anxiety and reaction times on the CRT. Thus, a potential explanation for the link between the CRT and maths anxiety could be that cognitive reflection is reduced in anxious participants, because they spend less time on the problems. In order to see if faster responding is necessarily associated with decreased performance on the CRT, we computed the correlations between performance on the CRT and reaction times separately for participants in the WM-load and no load conditions. In the no load condition there was no relationship between reaction times and performance on the CRT (*r*(29) = .10, *p* = .58). However, in the WM-load condition, there was a strong, significant relationship between reaction times and CRT performance (*r*(56) = .49, *p* < .001). This suggests that in the no load condition the link between CRT performance and anxiety cannot be explained by a tendency for anxious participants to rush through the problems. Specifically, although anxious participants tend to respond faster, this does not necessarily lead to performance decrements in all participants. However, under cognitive load, fast responses are strongly associated with reduced performance. Thus, rushing through problems might be particularly damaging for anxious participants when they are distracted, or when they are dealing with cognitively demanding tasks.

Finally, the fact that self-reported anxiety levels correlated strongly with AMAS scores confirms the ecological validity of the AMAS in predicting anxiety levels elicited by word problems including numerical content, which were presented in a low-stakes situation.

In our study we employed the DMT (a visuo-spatial task) as a secondary task, because this task has previously been found to increase participants’ tendency to rely on heuristic responses in the case of tasks with a heuristic-normative conflict [38,39]. However, it is worth noting that Ashcraft and Kirk [12] used verbal load as a secondary task in their classic experiment looking at the effects of maths anxiety and working memory load on maths performance. It is also common in the literature to attribute the effect of maths anxiety on maths performance to the WM-load imposed by anxious thoughts and ruminations (e.g., [13,43]). Based on these assumptions, it might seem more reasonable to employ a verbal secondary load to simulate the effects of maths anxiety. However, a study by [44] demonstrated, using a dual-task paradigm, that both verbal and visuo-spatial recall performance decreased as a direct function of increasing cognitive load, regardless of the nature of the cognitive load (i.e., whether it was spatial or verbal). That is, based on these findings, verbal and visuo-spatial activities seem to compete for a common, domain-general pool of resources. Thus, performance is affected in the same way, regardless of the type of cognitive load.

In our study, we collapsed the results across the low- and high WM-load conditions, as we found that performance across these two conditions was similar. The lack of difference between the low and high-load conditions was possibly the result of more variability in participants’ performance under cognitive load than in the control condition (i.e., the response patterns were more noisy). It is possible that this variability arose because participants used different strategies to maintain the dot patterns in their working memory, and some of these strategies were more successful than others. Although including a larger sample of participants could have helped to decrease the noise in the data, our results clearly show that employing a WM-load reduced performance on the CRT, which is also in line with Toplak et al. [23] who reported a relationship between participants’ performance on the CRT, and their WM-capacity.

Finally, we should note that our results do not exactly match the pattern reported by [12] who found that the arithmetic performance of anxious participants was disproportionally affected by a WM-load. In this respect, we should highlight that Ashcraft and Kirk [12] employed relatively easy mental arithmetic problems, which showed no effect of maths anxiety when they were performed without a cognitive load. By contrast, the CRT is affected by maths anxiety, even when it is administered alone. Thus, the interaction in Ashcraft and Kirk’s [12] experiment emerged because there was no difference between participants with high and low mathematical anxiety under no load, but highly anxious participants performed more poorly than non-anxious participants under cognitive load. By contrast, in our experiment the performance difference was already present in the no-load condition, and, thus, our results are not comparable to that of Ashcraft and Kirk [12] in this respect.

In sum, although our study does not provide definitive evidence that maths anxiety reduces cognitive reflection, as we did not include a manipulation of anxiety, it clearly demonstrates a link between the availability of working memory resources and cognitive reflection. That is, it provides a plausible explanation for how maths anxiety could potentially lead to reductions in cognitive reflection (i.e., through burdening working memory resources). Nevertheless, our results also show that the relationship between maths anxiety and performance on the CRT cannot simply be explained as arising from the WM-load imposed by anxious thoughts. Indeed, whereas WM-load was only associated with performance decrements on the CRT, maths anxiety was additionally linked to reduced response times, and reduced confidence in one’s own performance. These factors (i.e., faster responding and reduced confidence), in turn, could have independently contributed to the problems of anxious participants with solving the CRT.

### General discussion

In three experiments we tested the hypothesis that maths anxiety is associated with reduced cognitive reflection. As expected, we found that students with high maths anxiety performed worse on the cognitive reflection test than students with low levels of anxiety. This was the case both for secondary school (Experiment 2) and university students (Experiments 1 and 3). In fact, this effect was still present when we controlled for participants’ maths knowledge (in Experiment 1) or maths achievement (in Experiment 2), and test anxiety (in Experiments 1–3). Our last experiment also demonstrated a number of differences between low and high-anxiety participants in the way they tackled the problems.

Specifically, in line with Ashcraft and Kirk [12] and Mattarella-Micke et al. [17], we demonstrated that maths anxiety was associated with performance decrements, which were similar to the effect of working memory load. To the best of our knowledge, our Experiment 3 is the only one, apart from Ashcraft and Kirk [12], to demonstrate this through a dual-task manipulation, and it is the first one, which demonstrates this in the case of word problems including numbers.

Additionally, Experiment 3 also showed that mathematical anxiety was related to reduced levels of confidence in one’s own performance (see also [40]), and to a tendency to rush through problems (see also [13,16]), which resulted in shorter reaction times for anxious participants. Finally, we demonstrated that reduced confidence and shortened reaction times did not arise as the effect of hampered working memory resources.

These findings have several important implications. First, our results demonstrate that maths anxiety arises, and is associated with performance in low-stakes situations, and in the case of problems which do not include mathematical symbols (other than numerals). Second, we showed that the link between maths anxiety and cognitive reflection was partially mediated by reduced mathematical performance in anxious participants, as demonstrated by the mediating role of general maths knowledge and maths achievement in Experiments 1–2. Importantly, the first two studies also showed that maths anxiety was still associated with cognitive reflection once mathematical performance and test anxiety were controlled for. This is in line with earlier claims that maths anxiety is related to participants’ ability to reflect on their own performance, and to detect and correct their own errors (cf., [16]). This could have been the result of the reduced availability of cognitive resources, the reduced time spent on the problems and/or participants’ reduced confidence in their own performance.

As we described in the introduction, the CRT is considered to be more than just a numeracy test (cf., [21], although see [45]). Indeed, it correlates highly with tasks measuring risky decision-making, future discounting, and rational thinking [19,21,23]. Much research shows the negative effects of low numeracy on judgement and decision making [8,9], as well as on employability, mental and physical health, and criminal behaviour (e.g., [6,7]). The consistent relationship between maths anxiety and cognitive reflection that we demonstrated in our studies suggests that maths anxiety might also be related to individuals’ decision making skills. In fact, it is likely that maths anxiety is linked to individuals’ economic behaviour, as well as their understanding of risks. Although further investigations are needed to establish more specifically what are the decision-making situations that are most strongly affected by maths anxiety, our results suggest that besides the effects of numeracy, researchers of judgment and decision-making should also take into account the effects of maths anxiety.

It could be argued that our results that show a link between maths anxiety and cognitive reflection are unsurprising, given that maths anxiety has already been found to be related to an increased reliance on heuristics, reduced executive functioning, impaired mathematical performance, and a tendency to jump into conclusions. Nevertheless, it should be noted that these findings were demonstrated in separate studies, whereas we provide evidence for all of these claims, using a single task. Additionally, our results are particularly informative with regard to the claim that maths anxiety is linked to a tendency for participants to rely on simple heuristics. A unique property of the CRT is that, although the problems are open-ended, the vast majority of participants (over 90% in the studies that we reported above) produce either a correct response or a typical (incorrect) heuristic response. Although some researchers (e.g., [46]) argued that maths anxiety led to an increased reliance on using simple heuristic strategies, these claims were generally based on indirect evidence (i.e., that high-WM participants, who were supposedly more likely to rely on complex strategies than low-WM participants, were disproportionately affected by maths anxiety). The only exception is [47] who were able to provide more direct evidence for the use of simple heuristics under pressure. In Experiment 1 this evidence was based on participants’ self-reported solution strategies, and in Experiment 2 on participants’ performance on Luchins’ [48] water jug task, where complex and easy solution strategies could be discriminated. Our findings are unique in that we measured maths anxiety as an individual differences variable (instead of experimentally manipulating performance pressure). Additionally, the pressure scenario used by [47] was not specifically aimed at inducing anxiety about maths, but about participants’ performance in general. Moreover, we used a novel task, where heuristic and correct responses could be easily distinguished without having to rely on participants’ self-report, or having to prime participants by administering initial problems.

Nevertheless, there are a few limitations of our studies which have to be noted. Although we suggested that our results indicated a link between maths anxiety and decision making, we did not actually include any traditional measures of decision-making skills in our studies. Thus, further research is needed to confirm this prediction. We also did not manipulate maths anxiety directly, which could have provided definitive evidence for the causal link between maths anxiety and reduced performance on the CRT. Additionally, there is evidence that the link between maths anxiety and performance on maths problems is dependent on participants’ working memory capacity (e.g., [17]). However, we did not measure working memory capacity in our experiments.

Another potential criticism is that our measures of maths knowledge (in Experiment 1) and maths achievement (in Experiment 2) did not capture all the variance associated with individual differences in mathematical abilities, and thus, it is still possible that the link between maths anxiety and the CRT is in fact fully mediated by mathematical abilities. Although this is indeed possible, earlier studies showed that numeracy was only one of several factors that explain variance in the CRT (e.g., [25]), and in maths anxiety (e.g., [13]). Additionally, given the surface similarity between the correlates of maths anxiety and cognitive reflection (most importantly, the tendency to rely on simple, undemanding heuristics) it is unlikely that any measure of maths ability would fully explain the link between maths anxiety and cognitive reflection. Finally, our results suggest that there are several variables which could potentially mediate the link between maths anxiety and performance on problems with a numerical content (including general mathematical knowledge, self-confidence, and time spent on solving the problems). Thus, further studies are necessary to disentangle the causal links between all of these factors.

Finally, in each experiment we administered the AMAS before the CRT. This order of presentation (i.e., to administer the maths anxiety scale before the presentation of mathematical problems) is in line with the classic study by Ashcraft and Kirk [12]. Nevertheless, one could argue that drawing participants’ attention to their own anxiety could have affected their performance on the CRT. An alternative option could have been to reverse the presentation order. However, in that case one could have argued that a correlation between performance on the CRT and the AMAS was the result of participants’ reflecting on their perceived performance on the CRT when answering the questions in the AMAS. Another possibility would have been to use a random order of presentation. However, we believe that this is not appropriate in the case of an individual differences study. Overall, we do not see an ideal solution for the problem of presentation order. Nevertheless, it is worth considering this issue when interpreting the results.

Although several questions remain to be answered, we believe that our results contribute to a better understanding of the cognitive correlates of maths anxiety. Additionally, these findings are also relevant for researchers of decision-making and reasoning in terms of our understanding of what cognitive reflection is. Since its publication in 2005, Frederick’s paper on the CRT has been cited over 700 times. Indeed, the CRT is an extremely popular measure, as it only takes a few minutes to administer, and, at the same time, it is one of the best predictors of rational thinking and decision-making skills. Nevertheless, our results clearly show that rather than simply measuring a metacognitive trait, the CRT also has a significant mathematics component (see also [25,45]). Indeed, it is not only related to a person’s maths ability, but also to their levels of maths anxiety. Thus, our findings call for the urgent need for researchers to better understand the cognitive bases of “cognitive reflection”, and the tendency for people to jump into conclusions without a second thought.

## Endnotes

^a^Initially, 405 students participated in the experiment, but given the low proportion of male students in the sample, and the fact that previous studies reported gender differences in both the CRT [19] and in maths anxiety (e.g., [26]), we decided to restrict our analyses to the female participants.

^b^We also ran the analyses regarding accuracy and reaction times on the CRT with the inclusion of the three original CRT items only (which were administered as the first three items of the extended scale), and we obtained essentially the same results as with the extended scale. These results are reported in [49].

^c^We report this correlation collapsed across WM-load conditions, as the correlations in the two conditions were virtually the same.

## Competing interests

The authors declare that they have no competing interests.

## Authors’ contributions

KM and CP developed the concept for this work, and they designed the experiments. Data collection was performed by CB and KM. All authors contributed to data analysis and interpretation. KM drafted the manuscript, and CP provided critical revisions. All authors approved the final version of the manuscript for submission.
